# The Question of Raising the Official Rank of District Veterinarians in Austria

**Published:** 1898-06

**Authors:** 


THE QUESTION OF RAISING THE OFFICIAL RANK OF DISTRICT VETERINARIANS IN AUSTRIA.—An appeal by F. H. The state veterinarians of Austria have already attempted many times to improve their rank in the official classes by sending deputations and well-considered petitions to the proper departments. Each time the reception has been a courteous one; each time assurances have been given to the deputations that the difficult and beneficial work of the official veterinarians was known and recognized; each time they were given prospects of a reform in the present untenable relations of rank—every time the veterinarians have come away with great hopes and have announced the approach of better times, and every time these hopes have proved deceptive. If it is better in itself to feel compelled to beg for what belongs to us by right, and for that reason ought to be given us without a petition, it is all the more discouraging when no hearing is given to repeated petitions. In spite of this the veterinary organs combined again on the 5th of this month in an attempt to acquire their just rights. A delegation consisting of five district veterinarians made appeals before the Ministry of the Interior and that of Finance. Since that they handed in a petition in which they explained the real condition of affairs and begged that district veterinarians be enrolled in the X. and XI. class of public officials.

The first step in this proceeding did not seem favorable, for when the delegation appeared in the Interior Department they were met with the unpleasant information that in consequence of an unexpected conference of all the ministers all audiences on that day had to be declared off. Nothing daunted, however, they waited upon the accredited representatives of the absent ministers, as well as upon all the committees having to do with the subject. Everywhere their reception was a courteous one, and everywhere they received the assurance that the wishes of the veterinarians would receive careful consideration, and the matter would be settled as soon and as favorably as possible. Are we, then, to be put off, this time also, with mere promises? We do not wish to look at the dark side of the matter, but trusting to the justice of our cause and to the word of Councillor Sperk not only cherish new hopes, but await confidently that after a long period of mortifying disregard the hour will at last strike when a just reward will be given to honest service.—*Idem.*, February 10, 1898.

